# Sequential Activations of ChREBP and SREBP1 Signals Regulate the High-Carbohydrate Diet-Induced Hepatic Lipid Deposition in Gibel Carp (*Carassius gibelio*)

**DOI:** 10.1155/2023/6672985

**Published:** 2023-07-20

**Authors:** Yulong Gong, Longwei Xi, Yulong Liu, Qisheng Lu, Zhimin Zhang, Haokun Liu, Junyan Jin, Yunxia Yang, Xiaoming Zhu, Shouqi Xie, Dong Han

**Affiliations:** ^1^State Key Laboratory of Freshwater Ecology and Biotechnology, Institute of Hydrobiology, Chinese Academy of Sciences, Wuhan 430072, China; ^2^College of Advanced Agricultural Sciences, University of Chinese Academy of Sciences, Beijing 100049, China; ^3^The Innovative Academy of Seed Design, Chinese Academy of Sciences, Wuhan 430072, China; ^4^Hubei Hongshan Laboratory, Wuhan 430070, China

## Abstract

The present study investigated the sequential regulation signals of high-carbohydrate diet (HCD)-induced hepatic lipid deposition in gibel carp (*Carassius gibelio*). Two isonitrogenous and isolipidic diets, containing 25% (normal carbohydrate diet, NCD) and 45% (HCD) corn starch, were formulated to feed gibel carp (14.82 ± 0.04 g) for 8 weeks. The experimental fish were sampled at 2^nd^, 4^th^, 6^th^, and 8^th^ week. In HCD group, the hyperlipidemia and significant hepatic lipid deposition (oil red O area and triglyceride content) was found at 4^th^, 6^th^, and 8^th^ week, while the significant hyperglycemia was found at 2^nd^, 4^th^, and 8^th^ week, compared to NCD group (*P* < 0.05). HCD induced hepatic lipid deposition via increased hepatic lipogenesis (*acc*, *fasn*, and *acly*) but not decreased hepatic lipolysis (*hsl* and *cpt1a*). When compared with NCD group, HCD significantly elevated the hepatic sterol regulatory element binding proteins 1 (SREBP1) signals (positive hepatocytes and fluorescence intensity) at 4^th^, 6^th^, and 8^th^ week (*P* < 0.05). The hepatic SREBP1 signals increased from 2^nd^ to 6^th^ week, but decreased at 8^th^ week due to substantiated insulin resistance (plasma insulin levels, plasma glucose levels, and P-AKT^Ser473^ levels) in HCD group. Importantly, the hepatic carbohydrate response element binding protein (ChREBP) signals (positive hepatocytes, fluorescence intensity, and expression levels) were all significantly elevated by HCD-induced glucose-6-phosphate (G6P) accumulation at 2^nd^, 4^th^, 6^th^, and 8^th^ week (*P* < 0.05). Compared to 2^nd^ and 4^th^ week, the hepatic ChREBP signals and G6P contents was significantly increased by HCD at 6^th^ and 8^th^ week (*P* < 0.05). The HCD-induced G6P accumulation was caused by the significantly increased expression of hepatic *gck*, *pklr*, and *glut2* (*P* < 0.05) but not *6pfk* at 4^th^, 6^th^, and 8^th^ week, compared to NCD group. These results suggested that the HCD-induced hepatic lipid deposition was mainly promoted by SREBP1 in earlier stage and by ChREBP in later stage for gibel carp. This study revealed the sequential regulation pathways of the conversion from feed carbohydrate to body lipid in fish.

## 1. Introduction

Carbohydrates are widely applied in aquatic feeds as energy-supplying nutrients for their excellent price-performance ratio [1, 2]. However, many fish display a very limited ability in utilizing dietary carbohydrate [1, 3]. Consequently, a long-term excessive carbohydrate intake usually brings metabolic disorders, like disordered blood glucose [2, 4, 5], supraphysiological lipogenesis, and hepatic steatosis in various aquaculture species [6–8], including gibel carp (*Carassius gibelio*) [9]. In recent years, some studies focused on the metabolic syndrome caused by high-carbohydrate diet- (HCD-) induced over deposition of lipids [10, 11] and investigated the strategies for alleviating the HCD-induced metabolism disorders [12–14]. However, the dynamic changes of the conversion from feed carbohydrate to body lipid remains uncovered and whether existing sequential regulation pathways during the long-term HCD challenge is still poorly understood in the farmed fish.

In general, the lipogenesis process is mainly regulated by insulin signaling pathway [15]. After carbohydrate intake, the subsequent glucose influx triggers off insulin secretion, which abates endogenous glucose efflux and activates sterol regulatory element binding proteins 1 (SREBP1) to convert excess glucose into fatty acids via hepatic lipid anabolism [16, 17]. The regulation effects of SREBP1 in lipogenesis have been demonstrated in gibel carp [18], grass carp (*Ctenopharyngodon idella*) [3], and Atlantic salmon (*Salmo salar* L.) [19]. Notably, chronic overdeposition of lipids results in insulin resistance (high-insulin level while low-insulin efficiency) and impairs the insulin-SREBP1 signaling-mediated lipogenesis in mammalians [15, 20]. Accordingly, insulin resistance may be also established in farmed fish during the long-term HCD challenge. Importantly, previous investigations identified carbohydrate response element binding protein (ChREBP) as a key regulator in converting excess carbohydrate to lipid for long-term storage [21, 22]. ChREBP is a basic helix–loop–helix leucine-zipper transcription factor, playing crucial role in lipogenesis, and glycolysis under high-carbohydrate intake [23, 24]. Unlike SREBP1, the activation of ChREBP is promoted by carbohydrate-related metabolites, like glucose-6-phosphate (G6P) [25] and fructose 2,6-bisphosphate [26]. ChREBP and SREBP-1 showed overlapping but distinct roles in regulating postprandial hepatic lipogenesis [27]. Several studies indicated that ChREBP was involved in the carbohydrate-induced lipogenesis in aquaculture fish [3, 28]. Although SREBP1 and ChREBP seem to play overlapped roles in lipogenesis, the sequential responses and synergism of them during the long-term HCD intake remains elusive, which is of significance to the precise regulation of HCD-induced lipid overdeposition in farmed fish.

Gibel carp (*C. gibelio*) are widely cultured in China with an annual production of 2.78–million tons in 2021 [29]. As an omnivorous fish, gibel carp is a suitable model for its moderate carbohydrate-tolerance to investigate the metabolic alteration during a long-term HCD challenge [30]. In the present study, we evaluated the sequential changes of hepatic lipid deposition and lipogenesis regulation in the gibel carp fed with HCD for 8 weeks. The results demonstrated that HCD induced hepatic lipid deposition via increasing lipogenesis, which was mainly promoted by insulin-SREBP1 in earlier stage (4^th^ and 6^th^) and by G6P-ChREBP in later stage (6^th^ and 8^th^) during the 8 week experiment. Thus, we clarified a sequential regulation manner of the conversion from feed carbohydrate to body lipid in fish. These findings present sequential and precise targets for ameliorating the HCD-induced hepatic steatosis in cultured fish.

## 2. Materials and Methods

### 2.1. Experimental Diets

Two isonitrogenous (29.91% crude protein) and isolipidic (6.72% crude lipid) experimental diets were formulated, which contained 25% corn starch (normal carbohydrate diet, NCD) and 45% corn starch (HCD), respectively. The formulation and approximate chemical compositions of the experimental diets are shown in Table 1. The ingredients of each diet were thoroughly mixed through a 60 mesh sieve. Then, the ingredients were completely mixed and extruded into 3 mm diameter pellets by using a laboratory single-screw pelleter (SLR-45, Fishery Machinery and Instrument Research Institute, Chinese Academy of Fishery Science, Shanghai, China). Pellets were dried in an oven at 60°C and stored at 4°C before use.

### 2.2. Experimental Fish and Feeding Trial

The experimental gibel carp (*C. gibelio* var. CAS V) were obtained from the Institute of Hydrobiology, Chinese Academy of Sciences (Wuhan, Hubei, China). Four weeks prior to the feeding trial, gibel carp were acclimated in an indoor rearing system with fiber glass cylinder and fed to satiation twice a day at 8:30 and 16:30 with a commercial feed (Wuhan CP Aquatic Co., Ltd., Wuhan, China). After a 24 hr fasting, the fish of healthy appearance and uniform size (initial body weight: 14.82 ± 0.04 g) were randomly distributed into six indoor fiber glass tanks (water volume: 120 L) with 20 fish density for each tank. Triplicate tanks were assigned for each treatment. During the trial, fish were fed twice a day at 8:30 and 16:30 for 8 weeks. The water temperature was recorded daily and maintained at 27–29°C. Ammonia nitrogen (Ammonia-N), dissolved oxygen (DO), and pH were monitored every 3 days. The values showed that the concentration of Ammonia-N was below 0.3 mg/kg, the DO was 6.0–7.5 mg/L, and the pH was 6.8–7.3. The photoperiod was 12 hr light (8:00–20:00): 12 hr dark.

### 2.3. Sample Collection and Growth Performance Determination

Sample collections were conducted at 2^nd^, 4^th^, 6^th^, and 8^th^ week, respectively. For each sample collection, three fish in each tank were randomly selected and anesthetized with MS-222 (50 mg/L; Sigma Aldrich Co. LLC., St. Louis, MO, USA) at postprandial 4 hr. The body length and weight of the anesthetized fish were measured, then two of them were sampled blood from the caudal vein by heparinized syringe. The blood was centrifuged at 3,500 g for 10 min to obtain the plasma and stored at −80°C for further analysis. Immediately, the liver tissues were removed on ice. A small part of each liver tissues was fixed by 4% paraformaldehyde for histological staining and observation, while the rest was stored at −80°C for further analysis. The specific growth rate (SGR) and condition factor (CF) were calculated as follows:

(1) $$\begin{array}{c} \begin{array}{ccc} SGR\;\left(\%/d\right)\; & = & \;\left[Ln\;\left(final\;body\;weight\right)\;-\;Ln\;\left(initial\;body\;weight\right)\right]/days\;\times \;100;\\ CF\;\left(g/cm^{3}\right) & = & {whole\;body\;weight/\left(body\;length\right)}^{3}. \end{array} \end{array}$$

### 2.4. Total RNA Extraction, Reverse Transcription, and qPCR

Total RNA from the liver tissues was extracted by TRIzol Reagent (Ambion Life Technologies, Carlsbad, CA, USA) according to the product instructions. The quality and concentration of extracted total RNA were evaluated according to our previous methods [31]. The total RNA of liver tissues was reverse-transcribed with an M-MLV First Strand Synthesis Kit (Invitrogen, Shanghai, China) according to the product instructions. The obtained cDNA was stored at −20°C for qPCR analysis. The qPCR was conducted on LightCycle 480 II system (Roche, Basel, Switzerland). Samples were run in duplicates, and the relative expressions were calculated according to a published method [32]. The primers of qPCR analysis were designed with the National Center for Biotechnology Information (NCBI) primer BLAST service or from the previous study [18] and listed in Table 2.

### 2.5. Plasma and Tissue Biochemical Analyses

Plasma glucose levels were measured by Glucose Assay Kit with O-toluidine (Beyotime Biotechnology, China, S0201S). Hepatic and plasma triglycerides (TG) contents were measured using a Triglyceride Assay Kit (A110, Nanjing Jiancheng Bioengineering Institute, China). Plasma insulin level was measured using a commercial ELISA kit (H203, Nanjing Jiancheng Bioengineering Institute, China). Hepatic G6P content were measured using commercial Glucose-6-phosphate Assay Kit (S0185, Beyotime Biotechnology, China). All measurements were performed following the manufacturers' instructions.

### 2.6. Oil Red O Staining, Immunofluorescence Staining, and Imaging

The liver tissues initially fixed with 4% paraformaldehyde plus 20% sucrose overnight and sectioned to 8 *μ*m with a cryostat (Thermo Fisher Scientific, MA, USA). For oil red O (ORO) staining, the liver cryosections were stained with ORO to visualize the lipid droplets. Images were collected by Leica automatic digital slide scanner (Aperio VERSA 8, GER) and quantified with ImageJ (National Institutes of Health, USA). Immunofluorescence on the liver cryosections was performed according to a previous study [33]. Primary antibodies were anti-SREBP1 (Abcam, ab28481, and Rabbit, 1 : 200), anti-P-AKT^Ser473^ (Cell Signaling Technology, 4060, Rabbit, 1 : 200), and anti-CHREBP (Abcam, ab81958, Rabbit, 1 : 200), followed by Alexa Fluor 568 (Thermo Fisher Scientific, A11036, Goat anti rabbit, 1 : 1,000) or Alexa Fluor 488 (Cell Signaling Technology, 4412, Goat anti rabbit, 1 : 1,000) conjugated secondary antibody for visualization. Nuclei were stained by Hoechst 33342 (Thermo Fisher Scientific, H21492). Images were collected with Leica laser-scanning confocal microscope (SP8 DLS, GER), analyzed by Imaris Viewer (Oxford Instruments, UK), and quantified by ImageJ (National Institutes of Health, USA).

### 2.7. Statistical Analysis

All results are expressed as means ± SEMs (standard error of the mean). Normality was tested by 1-sample Kolmogorov–Smirnov test. Homogeneity of variance was examined by the Levene test. A two-tailed independent t test was used to evaluate the significant differences of measured parameters between two groups (data between NCD and HCD at each week). A one-way ANOVA with Duncan's multiple-range test was used to evaluate significant differences of measured parameters among several groups (data among four weeks of each treatment). Differences with *P* Values < 0.05 were considered significant. All statistical analyses were carried out and graphed with GraphPad Prism 8 (GraphPad Software, San Diego, CA, USA).

## 3. Results

### 3.1. Growth Performance, Plasma Glucose/TG, and Hepatic Lipid Deposition

**Figure 1:**
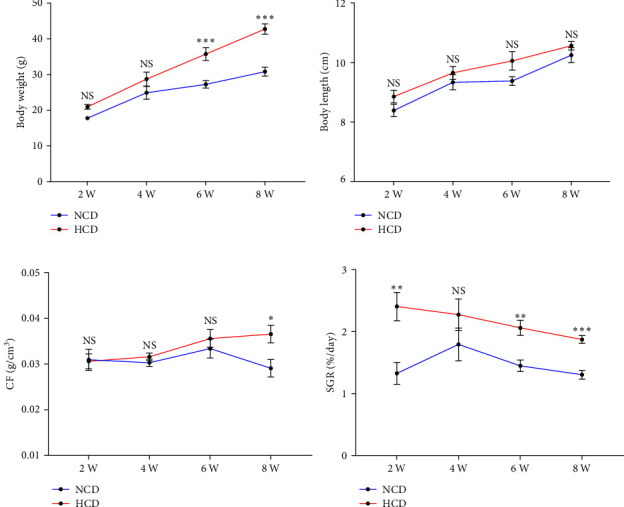
The growth performance of gibel carp fed with HCD and NCD during 8 weeks. (a) Body weight, (b) body length, (c) condition factor (CF), and (d) specific growth rate (SGR). Values are expressed as means ± SEMs, *n* = 9.  ^*∗*^,  ^*∗∗*^,  ^*∗∗∗*^Different from NCD:  ^*∗*^*P* < 0.05,  ^*∗∗*^*P* < 0.01, and  ^*∗∗∗*^*P* < 0.005, NS means no significant difference (two-tailed independent *t* test).

During the 8-week feeding trial, the body weight showed significantly increased at 6^th^ and 8^th^ week in HCD group (*P* < 0.05), while there was no significant difference between HCD and NCD groups at 2^nd^ or 4^th^ week (Figure 1(a)). The body length showed no significant difference between HCD and NCD groups from 2^nd^ to 8^th^ week (Figure 1(b)). The CF value of HCD group was significantly higher than that in NCD group only at 8^th^ week (*P* < 0.05) (Figure 1(c)). However, the SGR of HCD group was significantly increased at 2^nd^, 6^th^, and 8^th^ week, compared to NCD group (*P* < 0.05) (Figure 1(d)).

**Figure 2:**
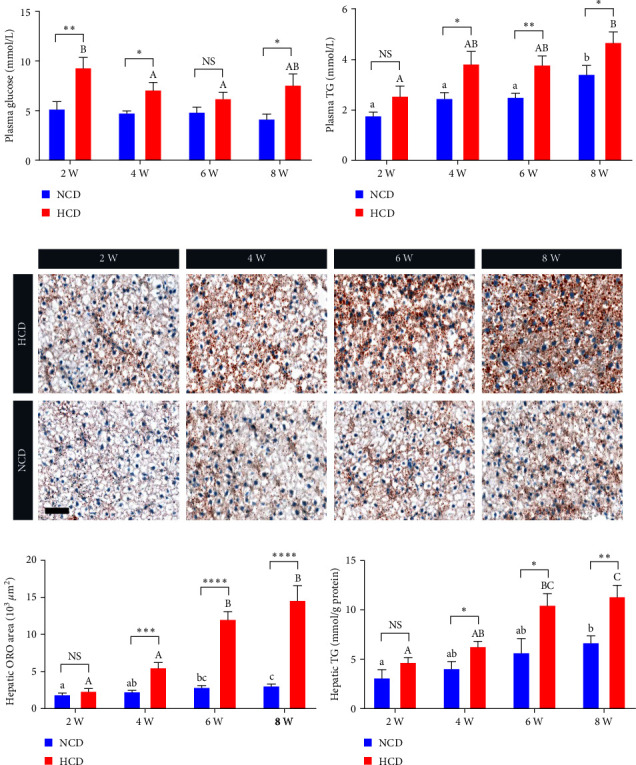
The plasma glucose, plasma triglyceride (TG) and hepatic lipid deposition levels of gibel carp fed with HCD and NCD during 8 weeks. (a) Plasma glucose level, (b) plasma TG level, (c) represented images of hepatic oil red O staining (scale bar = 50 *μ*m), (d) quantification of the hepatic oil red O area, and (e) hepatic TG content. Values are expressed as means ± SEMs, *n* = 6. Labeled means without a common letter differ among 2 W, 4 W, 6 W, and 8 W (lowercase for NCD and uppercase for HCD), *P* < 0.05 (one-way ANOVA, Duncan's post hoc test).  ^*∗*^,  ^*∗∗*^,  ^*∗∗∗*^,  ^*∗∗∗∗*^Different from NCD:  ^*∗*^*P* < 0.05,  ^*∗∗*^*P* < 0.01,  ^*∗∗∗*^*P* < 0.005, and  ^*∗∗∗∗*^*P* < 0.001, NS means no significant difference (two-tailed independent *t* test).

The plasma glucose levels of HCD group were significantly higher than those of NCD group at 2^nd^, 4^th^, and 8^th^ week (*P* < 0.05) (Figure 2(a)). Within the HCD group, the plasma glucose levels of 4^th^ and 6^th^ week displayed significantly decreased (*P* < 0.05), compared to that of 2^nd^ week (Figure 2(a)). The plasma TG levels of HCD group were significantly higher than those of NCD group at 4^th^, 6^th^, and 8^th^ week (*P* < 0.05) (Figure 2(b)). Within the HCD or NCD group, the plasma TG levels of 8^th^ week both showed significant increase, compared to those of 2^nd^, 4^th^, and 6^th^ week (*P* < 0.05) (Figure 2(b)). The ORO staining of hepatic sections showed that the lipid area of HCD group were significantly larger than those of NCD group at 4^th^, 6^th^, and 8^th^ week (*P* < 0.05) (Figures 2(c) and 2(d)). The hepatic TG content showed the similar change mode with the ORO staining results (Figure 2(e)). Within the HCD group, the hepatic lipid area and TG content of 6^th^ and 8^th^ week were significantly increased, compared to those of 2^nd^ and 4^th^ week (*P* < 0.05) (Figure 2(c)–2(e)). Besides, a mild but significant increase-trend in hepatic lipid area and TG content was also observed within the NCD group from 2^nd^ to 8^th^ week (Figure 2(c)–2(e)).

### 3.2. Expression of Hepatic Lipid Metabolism Genes

**Figure 3:**
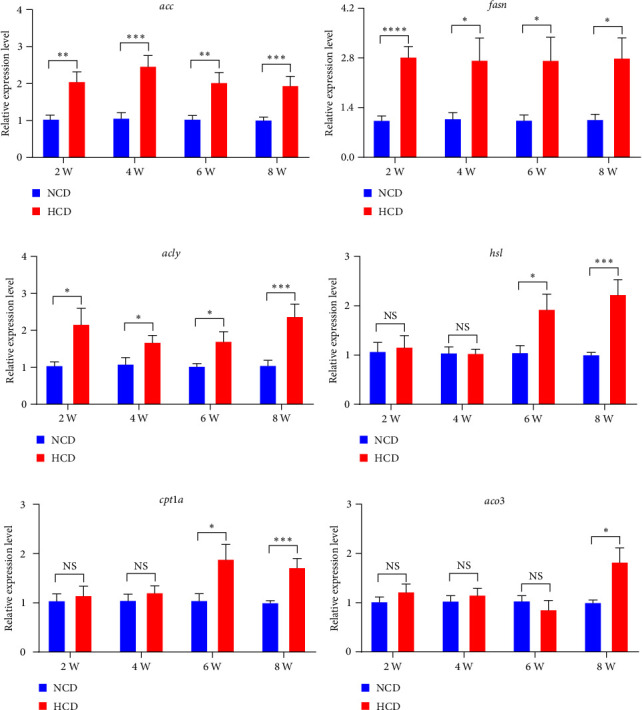
The expression levels of hepatic lipogenesis and lipolysis genes in gibel carp fed with HCD and NCD during 8 weeks. (a) Expression level of hepatic acetyl-CoA carboxylase (*acc*, gene), (b) expression level of hepatic fatty acid synthase (*fasn*, gene), (c) expression level of hepatic ATP-citrate lyase (*acly*, gene), (d) expression level of hepatic hormone-sensitive lipase (*hsl*, gene), (e) expression level of hepatic carnitine palmitoyl transferase 1a (*cpt1a*, gene), and (f) expression level of hepatic acyl-CoA oxidase 3 (*aco3*, gene). Values are expressed as means ± SEMs, *n* = 6.  ^*∗*^,  ^*∗∗*^,  ^*∗∗∗*^,  ^*∗∗∗∗*^Different from NCD:  ^*∗*^*P* < 0.05,  ^*∗∗*^*P* < 0.01,  ^*∗∗∗*^*P* < 0.005, and  ^*∗∗∗∗*^*P* < 0.001, NS means no significant difference (two-tailed independent *t* test).

The qPCR analysis of lipogenesis genes showed that the expression levels of hepatic acetyl-CoA carboxylase (*acc*), fatty acid synthase (*fasn*), and ATP-citrate lyase (*acly*) were all significantly increased in HCD group at 2^nd^, 4^th^, 6^th^, and 8^th^ week, compared to those in NCD group (*P* < 0.05) (Figure 3(a)–3(c)). However, the qPCR analysis of lipolysis genes showed that the expression levels of hepatic hormone-sensitive lipase (*hsl*) and carnitine palmitoyl transferase 1a (*cpt1a*) were significantly increased in HCD group at 6^th^ and 8^th^ week, while acyl-CoA oxidase 3 (*aco3*) significantly increased in HCD group only at 8^th^ week, compared to those in NCD group (*P* < 0.05) (Figure 3(d)–3(f)).

### 3.3. Evaluations of Hepatic Insulin-SREBP1 Signal and Insulin Resistance

**Figure 4:**
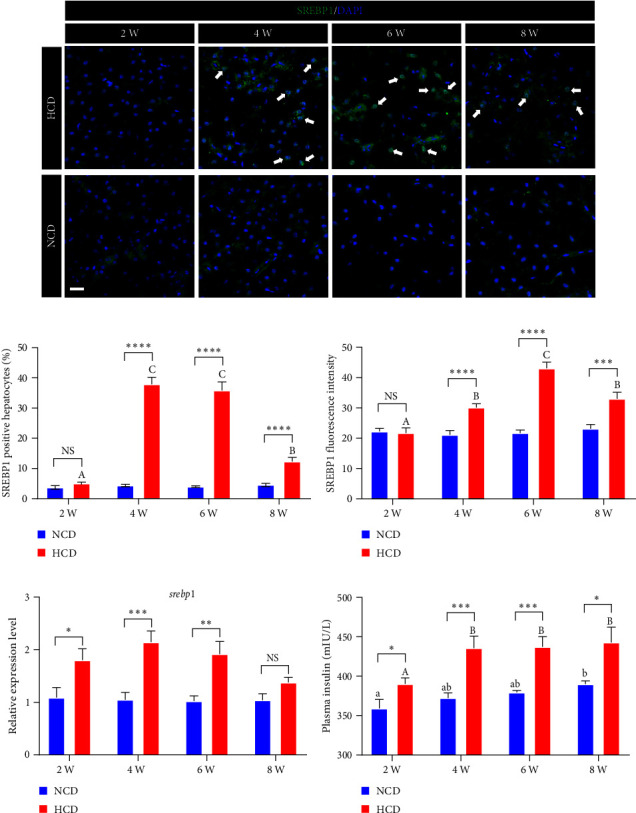
The expression and activation levels of hepatic insulin-SREBP1 signal in gibel carp fed with HCD and NCD during 8 weeks. (a) Represented SREBP1 (green) staining images (scale bar = 20 *μ*m), (b) percentage of nuclear SREBP1 positive hepatocytes, (c) average fluorescence intensity of nuclear SREBP1, (d) expression level of hepatic *srebp1*, and (e) plasma insulin level. Values are expressed as means ± SEMs, *n* = 6. Labeled means without a common letter differ among 2 W, 4 W, 6 W, and 8 W (lowercase for NCD, uppercase for HCD), *P* < 0.05 (one-way ANOVA, Duncan's post hoc test).  ^*∗*^,  ^*∗∗*^,  ^*∗∗∗*^,  ^*∗∗∗∗*^Different from NCD:  ^*∗*^*P* < 0.05,  ^*∗∗*^*P* < 0.01,  ^*∗∗∗*^*P* < 0.005, and  ^*∗∗∗∗*^*P* < 0.001, NS means no significant difference (two-tailed independent *t* test).

The immunostaining of hepatic SREBP1 showed that the percentage of SREBP1 positive hepatocytes and the fluorescence intensity of SREBP1 were significantly increased in HCD group at 4^th^, 6^th^, and 8^th^ week, compared to those in NCD group (*P* < 0.05) (Figure 4(a)–4(c)). Within HCD group, the percentage of SREBP1 positive hepatocytes and the fluorescence intensity of SREBP1 were significantly increased at 4^th^, 6^th^, and 8^th^ week, compared to those at 2^nd^ week (*P* < 0.05); however, they were both significantly decreased at 8^th^ week, compared to those at 6^th^ week (*P* < 0.05) (Figure 4(a)–4(c)). Besides, the expression levels of *srebp1* were all significantly increased in HCD group at 2^nd^, 4^th^, and 6^th^ week, but not 8^th^ week, compared to those in NCD group (*P* < 0.05) (Figure 4(d)). The plasma insulin levels of HCD group were significantly higher than those of NCD group at 2^nd^, 6^th^, 4^th^, and 8^th^ week (*P* < 0.05) (Figure 4(e)). Within the HCD group, the plasma insulin levels of 4^th^, 6^th^, and 8^th^ week displayed significantly increased compared to that of 2^nd^ week (*P* < 0.05), while there was no significant difference among 4^th^, 6^th^, and 8^th^ week (Figure 4(e)).

**Figure 5:**
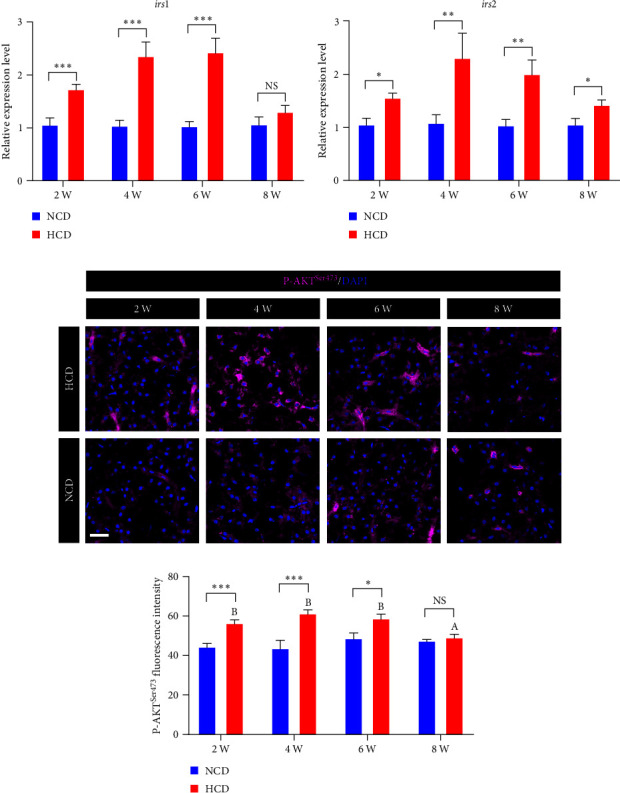
The expression and activation levels of hepatic insulin-AKT signal in gibel carp fed with HCD and NCD during 8 weeks. (a) Expression level of hepatic insulin receptor substrate 1-like (*irs1*, gene), (b) expression level of hepatic insulin receptor substrate 2-like (*irs2*, gene), (c) represented P-AKT^Ser473^ (Magenta) staining images (scale bar = 30 *μ*m), and (d) average fluorescence intensity of nuclear P-AKT^Ser473^. Values are expressed as means ± SEMs, *n* = 6. Labeled means without a common letter differ among 2 W, 4 W, 6 W, and 8 W (uppercase for HCD), *P* < 0.05 (one-way ANOVA, Duncan's post hoc test).  ^*∗*^,  ^*∗∗*^,  ^*∗∗∗*^,  ^*∗∗∗∗*^different from NCD:  ^*∗*^*P* < 0.05,  ^*∗∗*^*P* < 0.01,  ^*∗∗∗*^*P* < 0.005, and  ^*∗∗∗∗*^*P* < 0.001, NS means no significant difference (two-tailed independent *t* test).

The qPCR analysis of insulin receptor substrate (*irs*) genes showed that the expression levels of hepatic *irs1* and *irs2* were significantly increased in HCD group at 2^nd^, 4^th^, and 6^th^ week, but only *irs2* was significantly increased at 8^th^ week, compared to those in NCD group (*P* < 0.05) (Figures 5(a) and 5(b)). Meanwhile, the immunostaining of hepatic P-AKT^Ser473^ showed that the fluorescence intensity of P-AKT^Ser473^ was significantly increased in HCD group at 2^nd^, 4^th^, and 6^th^ week, but not 8^th^ week, compared to those in NCD group (*P* < 0.05) (Figures 5(c) and 5(d)). Within HCD group, the fluorescence intensity of P-AKT^Ser473^ was significantly decreased at 8^th^ week, compared to those at 2^nd^, 4^th^, and 6^th^ week (*P* < 0.05) (*P* < 0.05) (Figures 5(c) and 5(d)).

### 3.4. Expression and Activation of Hepatic G6P-ChREBP Signal

**Figure 6:**
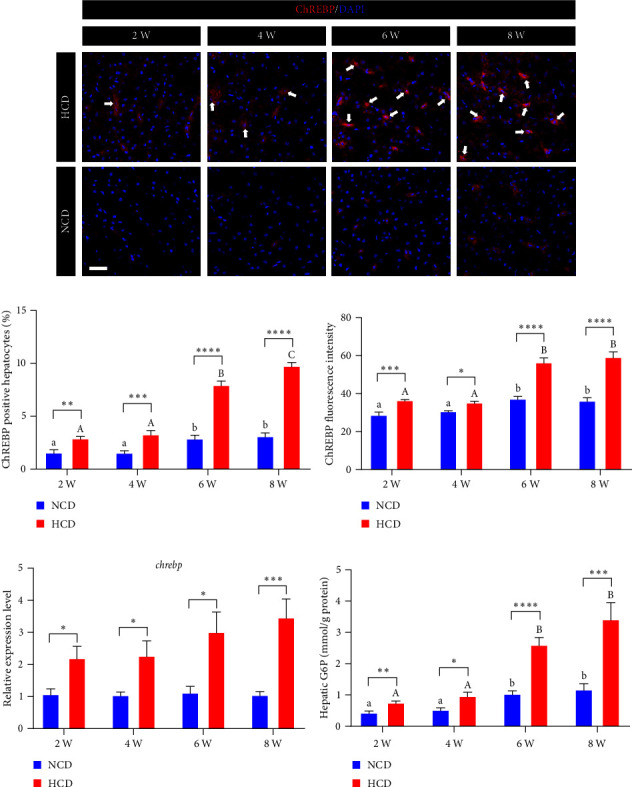
The expression and activation levels of hepatic G6P-ChREBP signal in gibel carp fed with HCD and NCD during 8 weeks. (a) Represented ChREBP (red) staining images (scale bar = 30 *μ*m), (b) percentage of nuclear ChREBP positive hepatocytes, (c) average fluorescence intensity of nuclear ChREBP, (d) expression level of hepatic *chrebp*, and (e) hepatic glucose-6-phosphate (G6P) content. Values are expressed as means ± SEMs, *n* = 6. Labeled means without a common letter differ among 2 W, 4 W, 6 W, and 8 W (lowercase for NCD, uppercase for HCD), *P* < 0.05 (one-way ANOVA, Duncan's post hoc test).  ^*∗*^,  ^*∗∗*^,  ^*∗∗∗*^,  ^*∗∗∗∗*^Different from NCD:  ^*∗*^*P* < 0.05,  ^*∗∗*^*P* < 0.01,  ^*∗∗∗*^*P* < 0.005,  ^*∗∗∗∗*^*P* < 0.001, NS means no significant difference (two-tailed independent *t* test).

The immunostaining of hepatic ChREBP showed that the percentage of ChREBP positive hepatocytes and the fluorescence intensity of ChREBP were all significantly increased in HCD group at 2^nd^, 4^th^, 6^th^, and 8^th^ week, compared to those in NCD group (*P* < 0.05) (Figure 6(a)–6(c)). Within HCD group, the percentage of ChREBP positive hepatocytes and the fluorescence intensity of ChREBP were significantly increased at 6^th^ and 8^th^ week, compared to those at 2^nd^ and 4^th^ week (*P* < 0.05); however, there was a further increase in the percentage of ChREBP positive hepatocytes at 8^th^ week, compared to those at 6^th^ week (*P* < 0.05) (Figure 6(a)–6(c)).Within NCD group, the percentage of ChREBP positive hepatocytes and the fluorescence intensity of ChREBP were also significantly increased at 6^th^ and 8^th^ week, compared to those at 2^nd^ and 4^th^ week (*P* < 0.05) (Figure 6(a)–6(c)). Besides, the expression levels of *chrebp* were all significantly increased in HCD group at 2^nd^, 4^th^, 6^th^, and 8^th^ week, compared to those in NCD group (*P* < 0.05) (Figure 6(d)). Moreover, the hepatic G6P contents of HCD group were all significantly higher than those of NCD group at 2^nd^, 6^th^, 4^th^, and 8^th^ week (*P* < 0.05) (Figure 6(e)). Within the HCD or NCD group, the hepatic G6P contents of 6^th^ and 8^th^ week displayed significantly increased compared to those of 2^nd^ and 4^th^ week (*P* < 0.05), respectively (Figure 6(e)).

### 3.5. Expression of Hepatic Glucose Metabolism Genes

**Figure 7:**
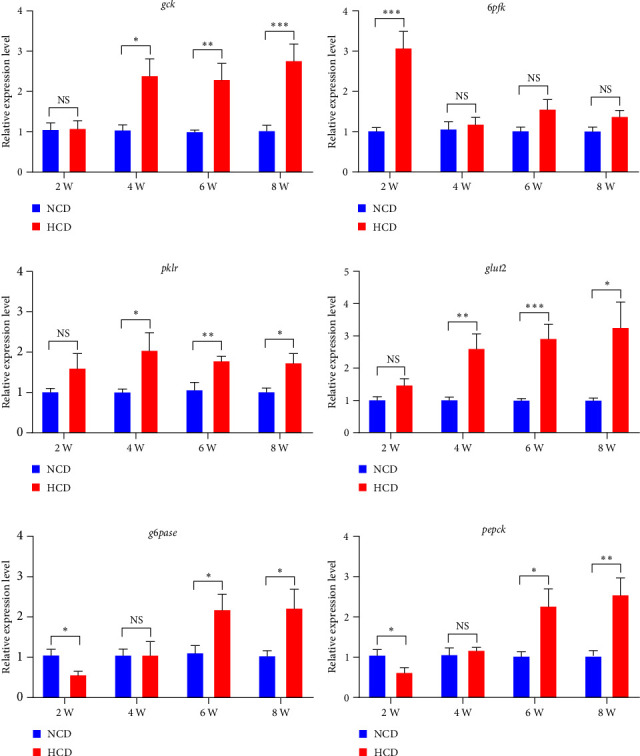
The expression levels of hepatic glycolysis and gluconeogenesis genes in gibel carp fed with HCD and NCD during 8 weeks. (a) Expression level of hepatic glucokinase (*gck*, gene), (b) expression level of hepatic 6-phosphofructokinase (*6pfk*, gene), (c) expression level of hepatic pyruvate kinase L/R (*pklr*, gene), (d) expression level of hepatic glucose transporter 2 (*glut2*, gene), (e) expression level of hepatic g6pase (*g6pase*, gene), and (f) expression level of hepatic phosphoenolpyruvate carboxykinase (*pepck*, gene). Values are expressed as means ± SEMs, *n* = 6.  ^*∗*^,  ^*∗∗*^,  ^*∗∗∗*^Different from NCD:  ^*∗*^*P* < 0.05,  ^*∗∗*^*P* < 0.01, and  ^*∗∗∗*^*P* < 0.005, NS means no significant difference (two-tailed independent *t* test).

The qPCR analysis of glycolysis and glucose transport genes showed that the expression levels of hepatic glucokinase (*gck*), pyruvate kinase L/R (*pklr*), and glucose transporter 2 (*glut2*) were all significantly increased in HCD group at 4^th^, 6^th^, and 8^th^ week, while 6-phosphofructokinase (*6pfk*) only significantly increased at 2^nd^ week, compared to those in NCD group (*P* < 0.05) (Figure 7(a)–7(d)). However, the qPCR analysis of gluconeogenesis genes showed that the expression levels of hepatic g6pase (*g6pase*) and phosphoenolpyruvate carboxykinase (*pepck*) were significantly decreased in HCD group at 2^nd^ week, while significantly increased in HCD group at 6^th^ and 8^th^ week, compared to those in NCD group (*P* < 0.05) (Figure 7(e)–7(f)).

## 4. Discussion

With the rapid development of artificial feeds, carbohydrates have become a basic dietary composition for aquatic animals. Fish have been thought to be inefficient in utilizing dietary carbohydrate, though carbohydrate is the most abundant energy-supplying nutrient in nature [1]. Interestingly, our recent study proposed that the conversion from glucose to lipids storage may be one of the efficient approaches for carbohydrate utilization in fish [3]. However, plenty of investigations demonstrated that excessive carbohydrate intake induced supraphysiological lipogenesis, that deteriorated to hepatic steatosis among aquaculture species [7, 8]. Therefore, depicting the comprehensive and precise regulation pathways of HCD-induced lipid deposition is of great significance for improving these negative effects caused by excessive dietary carbohydrate in fish. Accordingly, the present study investigated the sequential regulation manner of HCD-induced hepatic lipid deposition in gibel carp (*C. gibelio*). The results demonstrated that HCD promoted hepatic lipid deposition and lipogenesis mainly via insulin-SREBP1 in earlier stage (4^th^ and 6^th^) and via G6P-ChREBP in later stage (6^th^ and 8^th^) for gibel carp during the 8-week feeding experiment.

HCD induced lipid overdeposition from 6^th^ week via supraphysiological lipogenesis in gibel carp. Excessive carbohydrate intake leads to metabolic disorders, that is particularly true for fish [1, 34]. Increasing lipid storage is one the main approaches to cope with the high carbohydrate challenge in fish [35]. In the present study, we found that the obvious HCD-induced hepatic lipid accumulation and hyperlipemia were occurred from the 4^th^ to 8^th^ week, while the serve fatty liver started from the 6^th^ week under HCD treatment. The HCD-induced lipid accumulation in fish has been observed [6, 8, 9, 36]; however, here we demonstrated the dynamic changes of this process in gibel carp. We further showed the excessive lipid deposition mainly resulted from the supraphysiological lipogenesis but not decreased lipolysis in the liver of gibel carp. These results are similar to the previous studies in various of fish species [37–39]. In the present study, the lipolysis level even elevated at 6^th^ and 8^th^ week. The studies in largemouth bass (*Micropterus salmoides*) and zebrafish (*Danio rerio*) also indicated that the HCD-induced lipid accumulation was accompanied with activated lipolysis [10, 40]. However, studies in the yellow catfish (*Pelteobagrus fulvidraco*) and barramundi (*Lates calcarifer*) showed that dietary glucose and gelatinized wheat starch did not alter the lipolysis [6, 39]. We speculated this difference is due in part to the different carbohydrate sources and sampling time. Besides, studies in the largemouth bass and grass carp also demonstrated that HCD caused hepatic glycogen accumulation [3, 41], which is of interests for further investigations. In this study, we depicted the sequential process of HCD-induced hepatic lipid deposition and lipid metabolism in gibel carp.

Long-term HCD abated SREBP1 signal via insulin resistance in gibel carp. Insulin-SREBP1 is a key pathway in the motivation of lipogenesis in mammalians [15]. Plenty of studies showed that SREBP1 was also involved in the regulation of lipogenesis in aquaculture fish [37, 42], including gibel carp [18]. However, the activation and contribution mode of SREBP1 in HCD-induced lipogenesis have not been deciphered in the previous studies. In the present study, the expression and activation level of SREBP1 were elevated by HCD, those were consisted with previous studies in fish [10, 37, 42]. Notably, we found that the SREBP1 signal showed an obvious decrease from 6^th^ week to 8^th^ week, when we compared the dynamic changes of expression and activation level of SREBP1 within HCD group. This phenomenon has not been reported by previous studies. Persistent HCD usually leads to insulin resistance in mammalians [43, 44], which is characterized by fasting hyperglycemia, hyperinsulinemia, and insufficient phosphorylation of AKT [45]. Interestingly, the hepatic P-AKT^ser473^ level became insensitive to HCD, while the hepatic gluconeogenesis, plasma insulin, and plasma glucose levels were still higher in HCD group at 8^th^ week. These results indicated the insulin resistance was established in gibel carp after an 8-week HCD challenge. The similar results were also observed in rainbow trout (*Oncorhynchus mykiss*) [46]. Therefore, we proposed that the abated SREBP1 signal was due into the hepatic insulin resistance in this study. These results depicted the sequential regulating mode of SREBP1 in the HCD-induced lipogenesis of fish.

Although the SREBP1 signal was decreased by the insulin resistance at 8^th^ week, the hepatic lipogenesis and lipid deposition did not show any mitigating trend in HCD group. In mammalians, the elevated lipogenesis and nonalcoholic fatty liver disease also developed in the setting of insulin resistance [47, 48]. This process is speculated to be promoted by ChREBP [45], as it shares the same target lipogenesis genes with SREBP1 [22]. ChREBP and SREBP-1c have been thought to be both critical pathways to signal lipogenesis, those play overlapping but distinct roles in coordinating postprandial lipogenic [22, 27]. A previous review had postulated that glucose-responsive elements could be expected in the upstream regions of the lipogenesis genes in fish [35]. Interestingly, here we found that the ChREBP signal was significantly elevated by HCD from 2^nd^ to 8^th^ week in gibel carp. The expression and activation levels of ChREBP were even further increased at 6^th^ and 8^th^ week in HCD group. The increased gene expression levels of *chrebp* were also observed in yellow catfish [6], grass crap [3], and amur sturgeon (*Acipenser schrenckii*) [28] with carbohydrate challenges. However, the present study demonstrated the sequential activation changes of ChREBP in fish fed with HCD for the first time. The activation of ChREBP is modulated by glucose-related metabolites, especially G6P [25]. The intracellular G6P content was maintained homeostasis to be a stable pool by glycolysis and gluconeogenesis. Here we found that the hepatic G6P content displayed a similar change mode with the ChREBP expression and activation during the 8-week HCD trial in gibel carp, which indicated that the accumulated hepatic G6P pool promoted ChREBP activation. Moreover, we also observed the expression levels of *gck* were increased more than 2.28 fold to those in NCD group from 4^th^ to 8^th^ week, while the expression levels of *6pfk* showed no change between HCD and NCD groups. Thus, there was a fine-tuning in the glycolysis of gibel carp in HCD group. Since G6P is generated from glycolysis [49], produced by glucokinase and consumed by 6-phosphofructokinase, we speculate that this fine-tuning of glycolysis contributes to the accumulation of hepatic G6P. However, more solid evidences are required to confirm the relationships between G6P and this fine-tuning in further. Together, we revealed that the gradually amplified G6P-ChREBP signal dominated the later-stage hepatic lipogenesis and lipid accumulation in gibel carp fed with HCD.

**Figure 8:**
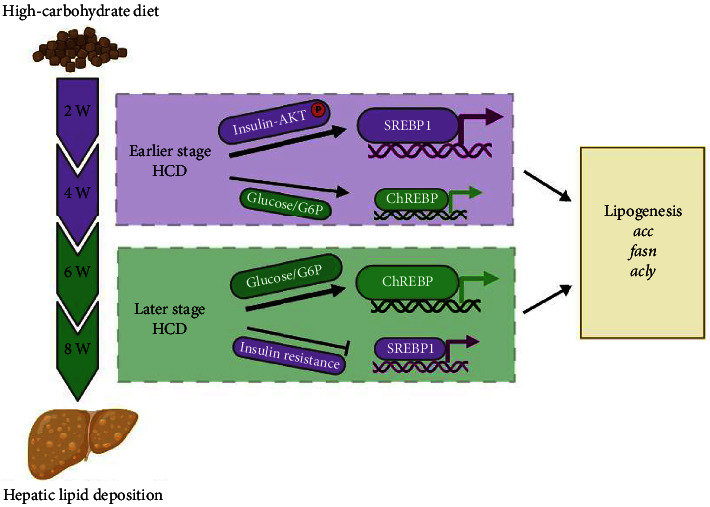
Proposed working model and regulation manner for ChREBP/SREBP1-mediated hepatic lipid deposition in gibel carp fed with HCD.

In conclusion, the present study demonstrated that HCD induced dynamic hepatic lipid deposition via increasing lipogenesis, that was mainly promoted by insulin-SREBP1 in earlier stage (4^th^ and 6^th^) while by G6P-ChREBP in later stage (6^th^ and 8^th^) for gibel carp during an 8-week feeding experiment (Figure 8). Therefore, we identified a sequential regulation manner for the conversion from feed carbohydrate to body lipid in fish. These findings present sequential and comprehensive insights for understanding the HCD-induced hepatic steatosis in aquaculture animals. Based on our findings, we propose that the feed carbohydrate level could be relatively higher and supplement with the SREBP1-targeting additives in the early-culturing stage; while the feed carbohydrate level could be relatively lower and insulin resistance-improving additives should be considered in the late-culturing stage. Besides, we recommend a pulsed carbohydrate level to avoid the health damage caused by persistent HCD intake.

## Figures and Tables

**Table 1:** Formulation and chemical composition of experimental diets (% dry matter).

Ingredient	Content (%)	
	NCD	HCD
White fish meal	15	15
Casein	24	24
Soybean oil	3	3
Fish oil	3	3
Corn starch	25	45
Vitamin premix^1^	0.39	0.39
Choline chloride	0.11	0.11
Mineral premix^2^	5	5
Cellulose	21.5	1.5
Carboxy methyl cellulose sodium	3	3
Proximate composition (%)		
Moisture	9.39	9.11
Crude protein	29.82	29.99
Crude lipid	6.81	6.62
Ash	7.42	7.23

^1^Vitamin premix (mg/kg diet): Vitamin B1, 20; Vitamin B2, 20; Vitamin B6, 20; Vitamin B12, 0.02; folic acid, 5; calcium patothenate, 50; inositol, 100; niacin, 100; biotin, 0.1; cellulose, 3522; Vitamin C, 100; Vitamin A, 110; Vitamin D, 20; Vitamin E, 50; Vitamin K, 10. ^2^Mineral premix (mg/kg diet): NaCl, 500.0; MgSO_4_·7H_2_O, 8,155.6; NaH_2_PO_4_·2H_2_O, 12,500.0; KH_2_PO_4_, 16,000; Ca(H_2_PO_4_)·2H_2_O, 7650.6; FeSO_4_·7H_2_O, 2,286.2; C_6_H_10_CaO_6_·5H_2_O, 1750.0; ZnSO_4_·7H_2_O, 178.0; MnSO_4_·H_2_O, 61.4; CuSO_4_·5H_2_O, 15.5; CoSO_4_·7H_2_O, 0.91; KI, 1.5; Na_2_SeO_3_, 0.60; corn starch, 899.7.

**Table 2:** Primer sequences for quantitative real-time PCR (qPCR) analysis.

Gene	Accession #	Forward primer (5′– 3′)	Reverse primer (5′– 3′)	Product size
*acc*	KF499584	GAGCTGTCTATCAGAGGAGACTTCA	GACGCTCGGCCTGCATCTTCT	139
*fasn*	KF511494	CCACACCATGGACCCACAGCT	CTGGGTCTTTACTGAAGGCCTCT	158
*acly*	KX898508	AGTTTGGCCACGCTGGAGCTTGT	CCCAGCTCATCGAAGCTCTTGG	112
*hsl*	MH536187	GAAGAGTGTTTCTATGCCTACT	CCGTGAGACATTGCCCTCAT	140
*cpt1a*	KX898509	GAAGCTCATCAGGCTGTGGCCTT	TTCCAGGAGTGAAGTCCGGAGAG	113
*aco3*	KX898510	TGTGGAGGACACGGTTACCTTGC	AGTTGCTGGTCTGCTGCAGAAGG	115
*srebp1*	KX898507	GGCCCTCTACTGCGTGGCACA	ACCACCATTTGGAGTGAGGGTCAC	194
*irs1*	XM026234327.1	CAACTACGCCCGTCCCTT	TCCGCCCTGATGACCTTA	168
*irs2*	XM026218815.1	CGGAAAGAATCTTGTAGTGG	TGCTCTGACGCATCATAAA	382
*chrebp*	XM026246198.1	CCGTCATAGATCCCGAAAG	TTACCATTGTCCTGGTTGGAGACTG	462
*gck*	KX898498	GAGGAGATGCGTAAGGTGGAGCT	TTCTCATACAGCTGATGTCCAGGGTT	167
*6pfk*	KX898500.1	ACACCGGATGCCGCAGAAGCA	TCGATCTCTCCGGTCACATACTCG	105
*pklr*	KX898502	GCATCTGTGTCTGCTGGACATCGA	TGAGAGCCGTGAGAGAAGTTCAGTC	144
*glut2*	KX898504	CTCGTGGATGAGCTACCTCAGCAT	CCCTGACTGAAGATCTCCGCCA	111
*g6pase*	KX898505	CCTTACTGGTGGGTCCATGAGACT	TGGGCCGGTCTCACAGGTCAT	90
*pepck*	KX898506	AGACAAACCCTCATGCCATGGCAAC	GGGTCTATGATGGGGCACTGG	226
*ef1α*	AB056104	GTTGGAGTCAACAAGATGGACTCCAC	CTTCCATCCCTTGAACCAGCCCAT	198

## Data Availability

The authors declare that all data supporting the findings of this study are available within the article, the source data provided with this paper.
